# The abnormal implicit memory to positive and negative stimuli in patients with current and remitted major depressive disorder: A systematic review and meta-analysis

**DOI:** 10.3389/fpsyt.2022.1043987

**Published:** 2023-01-10

**Authors:** Xingze Liu, Xiang Wang, Yao Liu, Feng Gao, Jie Xia, Jie Fan, Xiongzhao Zhu

**Affiliations:** ^1^Medical Psychological Center, The Second Xiangya Hospital, Central South University, Changsha, Hunan, China; ^2^Medical Psychological Institute of Central South University, Changsha, Hunan, China; ^3^National Clinical Research Center for Mental Disorders, Changsha, Hunan, China

**Keywords:** major depressive disorder, implicit memory, meta-analysis, stimuli types, memory bias

## Abstract

**Introduction:**

In patients with current major depressive disorder (*c*MDD) a general abnormal implicit memory has been reported. However, the elaborate function of implicit memory when processing stimuli with different emotions (i.e., positive, neutral, and negative) in current and remitted (*r*MDD) patients is unclear. The present review examines implicit memory’s general and elaborate in *c*MDD and *r*MDD patients.

**Methods:**

We conducted meta-analyses based on published studies meeting criteria in Web of Science, PubMed, and EMBASE databases between 1990 and July 2022. The full sample patients included *c*MDD = 601 and *r*MDD = 143.

**Results:**

Initial analysis of *c*MDD patients revealed a general implicit memory deficit. Subsequent subgroup analyses showed that the implicit memory performance to neutral stimuli is poorer in *c*MDD patients than controls, but recovered in *r*MDD patients; the deficient implicit memory to positive stimuli existed in *c*MDD and *r*MDD patients; the implicit memory performance to negative stimuli in *c*MDD patients is similar to controls but poorer in *r*MDD patients.

**Conclusion:**

These findings indicate that the negative bias in *c*MDD patients might compensate for the general implicit memory deficit. Together, the implicit memory to neutral stimuli could recover with remission, whereas still abnormal in processing positive and negative stimuli. These results suggested that the abnormal implicit memory to positive and negative information might be relevant to depression pathogenesis.

**Systematic review registration:**

https://www.crd.york.ac.uk/prospero, identifier CRD42020205003.

## 1. Introduction

Major depressive disorder (MDD) is a prevalent mental disorder (1, 2), accompanied by multiple cognitive abnormalities (3, 4). As one of the cognitive dysfunctions in MDD patients, the abnormality of implicit memory, which could be defined as unconscious or unintentional retrieval of past experience, has been broadly observed in patients who memorized more negative stimuli than healthy controls (5–7). This result is consistent with classical depression theories. For example, Beck et al. (8) suggested that MDD patients possessed stable and negative-biased representations of self-referential information like failure, loss, worthlessness, and hopelessness. Patients prefer to process various inputs toward negative experiences automatically once the negative representations stored in memory are activated (8–10). In other words, one activated negative memory node would automatically activate all the other associated negative nodes in memory (11–14). Such processing reflected a maladaptive memory pattern in MDD patients. As proposed in a study of Beevers (15), a cognitive vulnerability to depression is derived from the uncontrollable negative bias. If the stable and automatic bias cannot be controlled consciously, individuals may be more likely to develop depression. In contrast, if the implicit memory bias could be controlled consciously, it is possible to override the maladaptive pattern. However, the negative implicit memory bias was found in patients with current depression in most studies, whereas little was known whether the negative-bias was improved when they remitted from depression. Thus, a systematic and elaborate analysis of the implicit memory in patients with current and remitted major depressive disorder (i.e., *c*MDD and *r*MDD, correspondingly) could help recognize the stable pattern in patients’ implicit memory, and provide possible diagnosis and interventions.

As Graf and Schacter (16) have stated, the implicit memory is revealed when performance on a task is facilitated in the absence of conscious recollection. This facilitation is usually measured through the repetition priming effect (17, 18), which is referring to the facilitation effect of a pre-exposed object to an identical object (19). Previous studies investigated the implicit memory in MDD patients by manipulating the emotional types of stimuli. Generally, the stimuli could be categorized into three types (i.e., positive, neutral, and negative). For studies that adopted stimuli with multiple emotional types, participants were presented a series of stimuli with different emotions, and they were told to respond to some stimuli characteristics (e.g., pronounce stimuli or judge the emotional type of each stimulus). After the presentations, they were asked to recall or recognize the stimuli presented before. The percentage or number of recalls in each emotional type is regarded as indicators to evaluate patients’ implicit memory through comparisons with healthy controls; For studies only adopted neutral stimuli, there were always one or more regularities that were valid to improve performance but untold to participants. That is, the implicit memory occurs when the facilitation derived from the practice effect and benefits from the untold regularities. Importantly, these regularities are unconscious to participants after experiments (20). Therefore, the indicators to measure implicit memory are usually differences of reaction time or accuracy between trials with regularities and random. Most of these studies have observed that the implicit memory of *c*MDD patients was abnormal. However, this conclusion was inconsistent. For example, patients’ implicit memory bias to negative stimuli was found in many studies but not observed in other studies [e.g., see (21–23)]; Likewise, the impaired implicit memory to neutral regularities was controversial (24, 25). These in conclusions made it difficult to tell whether the general function of implicit memory was abnormal.

**FIGURE 1:**
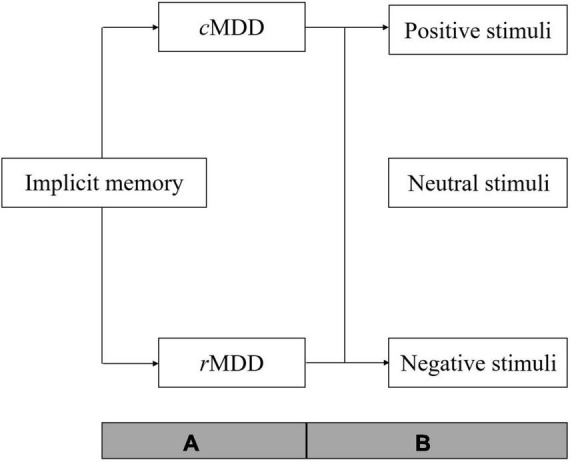
Over view of the analyses procedure. *c*MDD, patients with current major depressive disorder; *r*MDD, patients remitted from major depressive disorder; *A*, two initial meta-analyses were conducted to examine the general function of implicit memory in *c*MDD and *r*MDD patients; *B*, subgroup analyses of implicit memory to stimuli with different emotion types (positive, neutral, and negative) in *c*MDD and *r*MDD patients respectively.

In summary, one purpose of the present review is to examine the general function of implicit memory in *c*MDD patients. In addition, considering that previous studies were mainly focused on patients’ negative-biased implicit memory, we would categorize implicit memory into three sub-function according to the emotional types of stimuli (i.e., positive, neutral, and negative) for elaborately examining the abnormalities of implicit memory when processing different stimuli. Lastly, as proposed in theories mentioned above, the implicit memory abnormality should be a stable cognition pattern in MDD patients. Thus, we will examine whether the general function and three sub-function of implicit memory was abnormal in patients remitted from major depressive disorder (*r*MDD). As depicted in Figure 1, the meta-analysis was conducted with two steps. Firstly, we will conduct two initial analyses of *c*MDD and *r*MDD patients separately to examine the general function of implicit memory in each patient group; then, we divided the data of included studies into three subgroups according to stimuli types (i.e., positive, neutral, and negative) and conducted subgroup analyses to each subgroup of stimuli types in *c*MDD and *r*MDD patients separately.

## 2. Materials and methods

The primary design of present review was registered on PROSPERO (CRD42020205003), and was conformed to the Preferred Reporting Items for Systematic Reviews and Meta-Analyses (PRISMA) guidelines regarding evidence selection, quality assessment, evidence synthesis, and research reporting [Hutton et al. (26)].

### 2.1. Literature search and inclusion criteria

Literatures that were written in English between 1990 and April 2022 were primarily sourced in three databases: PubMed, Web of Science and EMBASE, using the following combination of key words: (“major depressive disorder” [All Fields] OR “major depression” OR “depression” [All Fields] OR “depressed” [All Fields] OR “MDD” [All Fields]) AND (“implicit” [All Fields] OR “automatic” [All Fields]) AND (“memory” [All Fields] OR “learning” [All Fields]). Two authors screened studies and extracted data independently, and any disagreement was resolved by discussion until a consensus was reached or by consulting a third author.

To be included in the analysis, the selective criteria for studies were: (1) MDD patients (mean age ≥ 18 years) diagnosed with the Diagnostic and Statistical Manual of Mental Disorders (DSM) (27) or International Classification of Diseases (ICD) (28), which are free from psychotic features, bipolar disorder, comorbid ADHD, or substance abuse; (2) studies matched depressed patients with healthy controls; (3) studies using at least one psychological paradigm to measure the implicit memory; (4) sufficient data was reported to estimate effect sizes (e.g., mean and standard deviation or standard error data) for both groups; and (5) only case-control should be included.

### 2.2. Study selection and data extraction

All identified titles and abstracts were independently assessed for eligibility by two authors (XzL and YL) using a pilot form. Any disagreement in selected studies was resolved by discussion, and the arbitration of the third author (XW). One reviewer (XzL) conducted the full-text reviews of the reports and extracted the data into the structured forms. Then, another reviewer (XW) verified its completeness and accuracy. The included studies were attentively studied, the information collected is listed below: the location, author, and publication year of study; the age, gender, clinical data of participants, and outcomes of experiments (mean value and corresponding standard deviation of each experiment condition). For the outcome indicators, reaction time, numbers/percentage of recall, and fixed duration time of eye-tracking were collected.

### 2.3. Quality assessment

We applied the Newcastle-Ottawa Scale (29) to assess methodological quality of included studies in view of its’ comparable comprehensive evaluate contents for case-control studies. The scores of 7-9, 4-6 and ≤ 3 in the Newcastle-Ottawa Scale are representative of high, moderate, and low quality in case-control studies accordingly. This part was performed by two investigators (XzL and XW). Any disagreements were resolved by consensus discussion with all authors.

### 2.4. Statistical analysis

As stated above, the analyses were conducted with the following steps: (1) two initial meta-analyses to data of *c*MDD and *r*MDD patients to examine the general function of implicit memory, (2) subgroup analyses of implicit memory to stimuli with different emotion types (i.e., positive, neutral, and negative) in *c*MDD and *r*MDD patients separately.

Stata version 12 was applied for data analysis. We calculated standardized mean differences (SMDs) and 95% confidence intervals (CIs) indicating the difference between patients and healthy controls. When experiment is conducted to same participants repeatedly (i.e., prior-treatment and post-treatment), only the performance in prior-treatment is included for the analysis. The magnitude of SMDs indicates: (0-0.2) = negligible effect, (0.2-0.5) = small effect, (0.5-0.8) = moderate effect, (0.8 +) = large effect (30). Heterogeneity is estimated with the I^2^ statistic. I^2^ statistic of 25, 50 and 75% were generally interpreted as small, moderate and high heterogeneity, respectively (31). In order to address heterogeneity, the random effect model is used. When the heterogeneity is high, we would conduct leave-one-out sensitivity analyses and random-effects meta-regression analyses to examine individual moderators, and if more than one moderator significantly predicted variance in effect size, we examined the moderators jointly as predictors.

## 3. Results

### 3.1. Included studies and quality assessment

#### 3.1.1. Included studies

**FIGURE 2:**
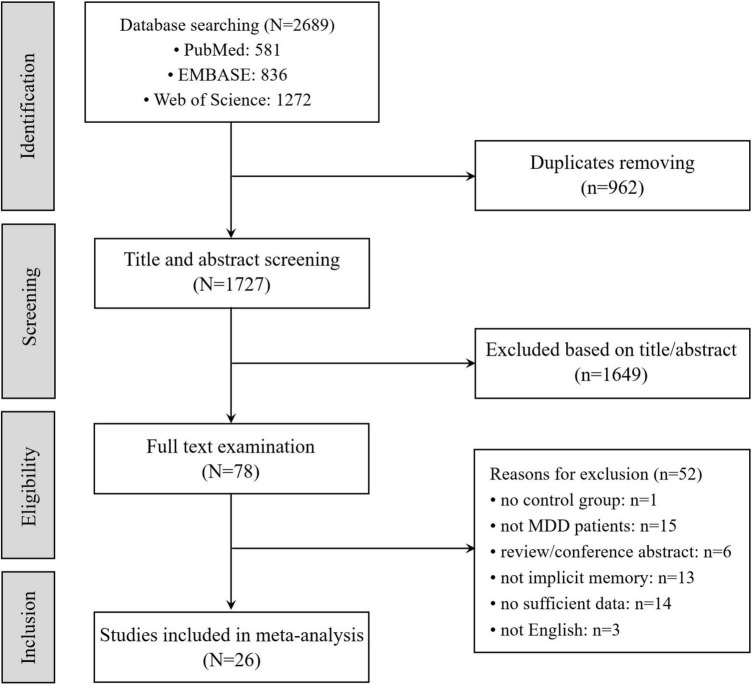
Flowchart of the trial selection process.

The procedure of literature searching is depicted in Figure 2. It was conducted through four steps (*Identification*, *Screening*, *Eligibility*, and *Inclusion* with two authors independently. In *Identification*, we searched three data bases (PubMed, Web of Science and EMBASE) with key words: (“major depressive disorder” [All Fields] OR “major depression” OR “depression” [All Fields] OR “depressed” [All Fields] OR “MDD” [All Fields]) AND (“implicit” [All Fields] OR “automatic” [All Fields]) AND (“memory” [All Fields] OR “learning” [All Fields]). There were 2,689 studies in total collected in the three data bases (581 studies in PubMed, 836 studies in EMBASE, 1,272 studies in Web of Science) and 1,727 studies after removed 962 duplicated studies from these data bases. In *Screening*, 1,649 of 1,727 studies are excluded because of their irrelevant title or abstract. In *Eligibility* and *Inclusion*, 26 studies are included in meta-analysis after removed 52 studies that did not meet standards.

There were total of 744 patients (143 *r*MDD and 601 *c*MDD) and 790 healthy controls included in 26 studies, and a group of healthy controls matched with patients in each included study. For *c*MDD patients, the sample size in these studies was ranged from 10 to 67, with mean age ranged from 23.5 ± 4.6 to 72.4 ± 9.0 years; for *r*MDD patients, the sample size ranged from 20 to 93, the mean age was ranged from 21.57 ± 1.43 to 36.2 ± 9.6 years; for healthy controls, the sample size and mean age range were 20 to 35 and 22.10 ± 1.95 to 35.1 ± 8.9 years. The detail characteristics of included studies were summarized in Table 1.

**TABLE 1:** Characteristic of included studies.

References	Patient					Control		Stimuli type
	*N* (F/M)	Age (years)	Diagnostic	Status	Depression severity	*N* (F/M)	Age (years)	
Elliott and Greene (32)	10 (NR)	31.5 ± NR	RDC	*cMDD*	HRSD: 27.3 (range = 20 ∼ 36)	10 (NR)	31.9 ± NR	Positive, Neutral, Negative
Bazin et al. (33)	23 (16/7)	43.22 ± 13.07	DSM-III-R	*cMDD*	BDI: 21.3 ± 6.30MADRS: 35.26 ± 4.92	37 (25/12)	45.24 ± 14.41	NC
Danion et al. (22)	30 (19/11)	41.2 ± 11.5	DSM-III-R	*cMDD*	HAMD-21: 29.5 (range = 18∼49)MADRS: 33.4 (range = 16∼48)	30 (19/11)	41.5 ± 11.6	Positive, Neutral, Negative
Ilsley et al. (34)	15 (6/9)	47.3 ± 16.2	DSM-III-R	*cMDD*	HRSD: 26.4 ± 5.9	15 (4/11)	43.6 ± 12.3	Positive, Negative
Bazin et al. (21)	23 (16/7)	43.2 ± 13	DSM-III-R	*cMDD*	BDI: 21.3 ± 6.30MADRS: 35.26 ± 4.92	37 (25/12)	44.2 ± 14	Positive, Negative
Watkins et al. (35)	67 (52/15)	NR	DSM-IV	*cMDD*	BDI-I: NR	67 (52/15)	NR	Positive, Neutral, Negative
Ellwart et al. (36)	36 (28/8)	42.06 ± 12.08	DSM-IV	*cMDD*	FDD: 39.9 ± 7.99	36 (26/10)	42.47 ± 12.76	Positive, Neutral, Negative
Tarsia et al. (23)	18 (8/10)	43.11 ± 8.93	DSM-IV ICD-10	*cMDD* *cMDD*	BDI-I: 25.67 ± 7.31	18 (10/8)	38.00 ± 9.91	Positive, Neutral, Negative
Aizenstein et al. (37)	11 (6/5)	68.70 ± 6.00	DSM-IV	cMDD	HAMD-17: 18.5 ± 4.8	11 (6/5)	71.3 ± 6.26	Neutral
Lim and Kim (38)	26 (NR)	36.53 ± 13.58	DSM-IV	cMDD	BDI-I: 24.20 ± 11.72	33 (16/17)	33.76 ± 7.96	Positive, Neutral, Negative
Rinck and Becker (39)	27 (NR)	23.5 ± 4.6	DSM-IV	cMDD	FDD: 25.3 ± 7.7	55 (NR)	21.4 ± 2.4	Positive, Neutral, Negative
Naismith et al. (40)	21 (NR)	53.9 ± 11.8	DSM-IV	cMDD	HAMD-17: 21.7 ± 4.4	21 (NR)	50.8 ± 11.7	Neutral
Lamy et al. (41)	18 (7/11)	38.8 ± 12.9	DSM-IV	cMDD	BDI-I: 24.8 ± 10.4	18 (7/11)	37.6 ± 11.8	Neutral
Vázquez et al. (7)	35 (26/9)	39.6 ± 12.2	DSM-IV	cMDD	BDI: 26.3 ± 1.1	36 (15/21)	30.4 ± 7.4	Positive, Neutral, Negative
Exner et al. (42)	26 (20/6)MEL 9 (4/5)Non-MEL	33.0 ± 10.0 35.0 ± 10.5	DSM-IV	cMDD	BDI MEL: 26.7 ± 10.3 BDI Non-MEL: 21.5 ± 8.3 HAMD-17 MEL: 22.1 ± 4.4 HAMD-17 Non-MEL: 16.3 ± 4.5	26 (18/8)	33.0 ± 8.9	Neutral
Ridout et al. (43)	16 (11/5)	43.7 ± 11.3	ICD-10	cMDD	BDI-I: 31.8 ± 1.8	18 (14/4)	39.3 ± 8.8	Positive, Neutral, Negative
Pedersen et al. (44)	20 (10/10)	36.2 ± 9.6	DSM-IV	rMDD	BDI: 10.9 ± 6.5 HDRS: 3.9 ± 2.8	20 (10/10)	35.1 ± 8.9	Neutral
Naismith et al. (45)	19 (14/5)	56.1 ± 9.8	DSM-IV	cMDD	HAMD-17: 21.6 ± 4.2	20 (14/6)	50.6 ± 11.9	Neutral
Elderkin-Thompson et al. (46)	32 (NR)	NR	DSM-IV	cMDD	BDI-I: 26.6 ± 8.2 HAMD-17: 18.3 ± 3.4	45 (NR)	NR	Neutral
Romero et al. (47)	30 (24/6)	21.57 ± 1.43	DSM-IV	rMDD	BDI-II: 6.13 ± 3.39	30 (24/8)	22.10 ± 1.95	Positive, Neutral, Negative
Callahan et al. (5)	19 (15/4)	72.4 ± 9.0	DSM-IV	cMDD	GDS: 16.1 ± 3.8	28 (21/7)	72.1 ± 8.1	Positive, Neutral, Negative
Mörkl et al. (48)	44 (31/13)	39.27 ± 11.59	DSM-IV	cMDD	HRSD: 22.70 ± 4.90 BDI-I: 25.02 ± 9.23	44 (29/15)	40.5 ± 14.9	Neutral
Nemeth et al. (49)	28 (23/5)	49.22 ± 10.88	DSM-IV	cMDD	HRSD: 18.15 ± 5.21	28 (18/10)	46.83 ± 10.85	Positive, Neutral, Negative
Romero et al. (6)	38 (30/8)	26.79 ± 8.53	DSM-IV	cMDD	BDI-II: 26.74 ± 9.55	40 (31/9)	25.45 ± 7.93	Positive, Negative
Janacsek et al. (24)	10 (6/4)	47.90 ± 10.77	DSM-IV-TR	cMDD	BDI: 33.70 ± 8.68	10 (7/3)	44.58 ± 16.25	Neutral
Brian et al. (50)	93 (73/20)	23.17 ± 3.40	DSM-IV-TR	rMDD	HDRS: 3.63 ± 4.14	35 (20/5)	22.40 ± 3.08	Positive, Neutral, Negative

DSM-x, Diagnostic and Statistical Manual of Mental Disorder; ICD-x, International Classification of Diseases; cMDD, patients with current depression; rMDD, patients remitted from depression; HAMD-17/21/x, Hamilton Depression Rating Scale (17-/21-item/unstated version); BDI-I/13/II, Beck depression inventory (I-/II-version); MADRS, Montgomery-Asberg Depression Rating Scale; GDS, Geriatric Depression Scale; FDD, questionnaire for depression diagnosis (German version); QSD, severity of depression questionnaire; NR, not report; NC, not control.

#### 3.1.2. Quality assessment

As illustrated in Table 2, the average of the total score in NOS was 6.12. 17 studies showed moderate methodological quality, and nine of the rest are with high methodological quality.

**TABLE 2:** Quality assessment of included studies.

References	Selection				Comparability	Exposure		Total score^a^
	Case definition	Representa-tiveness of case	Selection of controls	Definition of controls	Comparability of cases and controls	Ascertain-ment of exposure	Same ascertain-ment for case/ Control	
Elliott and Greene (32)	✩	✩	✩		✩ ✩		✩	6
Bazin et al. (33)	✩	✩		✩	✩		✩	5
Danion et al. (22)	✩	✩	✩	✩	✩		✩	6
Ilsley et al. (34)	✩	✩	✩		✩		✩	5
Bazin et al. (21)	✩		✩	✩	✩		✩	5
Watkins et al. (35)	✩	✩	✩	✩	✩		✩	6
Ellwart et al. (36)	✩	✩	✩	✩	✩		✩	6
Tarsia et al. (23)	✩	✩		✩	✩		✩	5
Aizenstein et al. (37)	✩	✩	✩	✩	✩ ✩		✩	7
Lim and Kim (38)	✩	✩		✩	✩ ✩		✩	6
Rinck and Becker (39)	✩	✩	✩	✩			✩	5
Naismith et al. (40)	✩	✩	✩	✩	✩ ✩		✩	7
Lamy et al. (41)	✩	✩		✩	✩		✩	5
Vázquez et al. (7)	✩	✩		✩	✩		✩	5
Exner et al. (42)	✩	✩	✩	✩	✩ ✩		✩	7
Ridout et al. (43)	✩	✩		✩	✩ ✩		✩	6
Pedersen et al. (44)	✩		✩	✩	✩ ✩		✩	6
Naismith et al. (45)	✩	✩		✩	✩		✩	5
Elderkin-Thompson et al. (46)	✩	✩	✩	✩	✩		✩	6
Romero et al. (47)	✩	✩	✩		✩✩		✩	6
Callahan et al. (5)	✩	✩	✩	✩	✩ ✩	✩	✩	8
Mörkl et al. (48)	✩	✩	✩	✩	✩ ✩		✩	7
Nemeth et al. (49)	✩	✩	✩	✩	✩ ✩		✩	7
Romero et al. (6)	✩	✩	✩	✩	✩ ✩		✩	7
Janacsek et al. (24)	✩	✩	✩	✩	✩✩	✩	✩	8
Brian et al. (50)	✩	✩	✩	✩	✩ ✩		✩	7

A study can be awarded a maximum of 1 star for each item within the selection and exposure categories; a maximum of two stars can be given for comparability (✩ means yes, a total score of 7-9 indicates a high methodological quality, 4-6 indicates a moderate quality, and ≤3 indicates a low quality). ✩ ✩ Means studies met all two standards of the item.

In *Selection* part, the diagnostic criteria of all patients were DSM, ICD, or RDC. MDD patients and healthy controls in most of the included studies had corresponding representativeness; 19 studies recruited healthy controls from the community, and 23 studies defined healthy controls without any mental disorder history. In *Comparability* part, patients and controls in 25 studies matched age and/or other factors (e.g., gender, education, and IQ) to ensure the comparability of groups. There were only 2 studies that met the ascertainment of exposure criteria in *Exposure* part.

### 3.2. Meta-analysis results

#### 3.2.1. Initial meta-analysis

**FIGURE 3:**
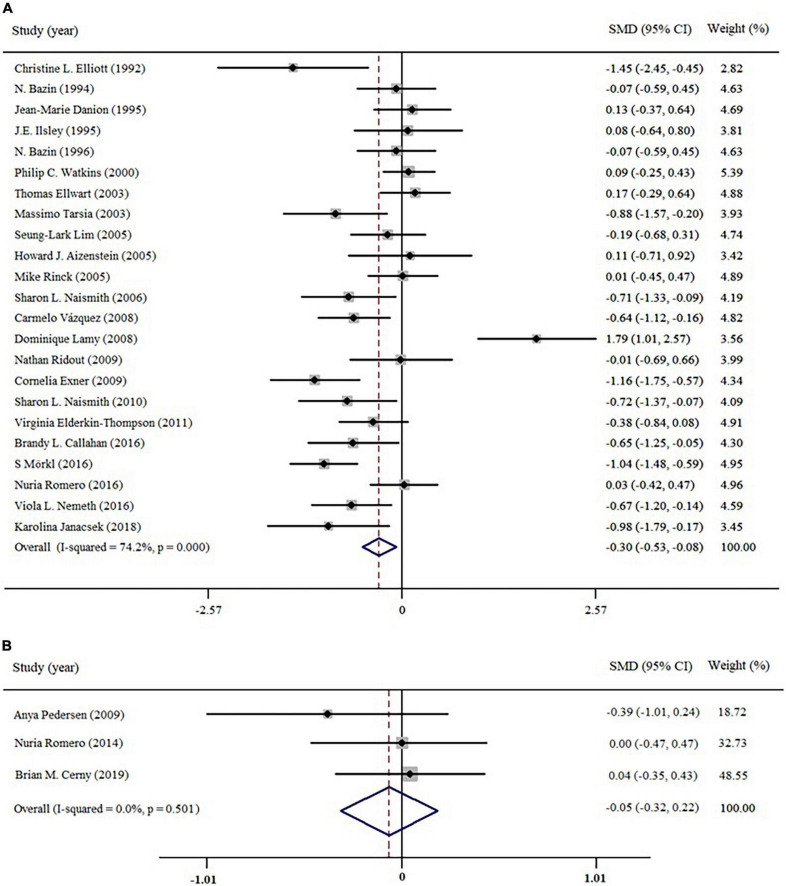
Forest plots of effect estimates of the general function of implicit memory in patients **(A)** current depression; **(B)** remitted depression compared to controls.

All included studies were divided into two data sets according to the depression status of patients (*c*MDD and *r*MDD). We then conducted two initial analyses to examine the general function of implicit memory in *c*MDD and *r*MDD patients (see Figure 3). For *c*MDD patients, the general function of implicit memory was significantly poorer than healthy controls (Effect size = −0.30; 95% CI: −0.53 to −0.08; *p* < 0.05), with the *I*^2^ of 74.2%. Meanwhile, the Egger’s test revealed no evidence for a publication bias (Egger’s intercept = −1.21; 95% CI: −4.98 to 2.31, *p* = 0.46); for *r*MDD patients, the implicit memory was not significantly different between patients and controls for Effect size = −0.05, 95% CI: −0.32 to −0.22; *p* = 0.71, with *I*^2^ of 0.0%. The Egger’s test showed no publication bias to studies of *r*MDD patients (Egger’s intercept = −1.36; 95% CI: −18.27 to 11.39, *p* = 0.21).

On account of the high heterogeneity (*I*^2^ = 74.2%) in studies of current depression, we then applied Galbraith graph to trace studies that possibly contributed to the heterogeneity (see Supplementary material). The graph indicated that there were five studies (32, 35, 41, 42, 48) primarily contributed the high heterogeneity. After removing these studies, the heterogeneity was decreased from 74.2 to 40.2%. Therefore, subsequent analyses would exclude the five studies.

#### 3.2.2. Sub-group analyses

In this phase, we conducted three subgroup analyses according to the stimuli types (i.e., positive, neutral, and negative) in *c*MDD and *r*MDD separately. The data of *c*MDD patients was extracted from 18 studies, and the rest of 3 studies were *r*MDD patients. In studies of *c*MDD, one did not categorize the emotion types of stimuli (33), three only manipulated positive and negative stimuli (6, 21, 34), and 5 studies only adopted neutral stimuli (24, 37, 40, 45, 46). Two studies of rMDD patients only adopted positive and neutral stimuli severally [(50); Pedersen et al. (44)].

For *c*MDD patients, the implicit memory to neutral (19 studies) and positive stimuli (11 studies) were significantly poorer than controls for Effect size = −0.56, 95% CI = −0.86∼−0.25, *p* < 0.001, *I*^2^ = 84.3% and Effect size = −0.60, 95% CI = −1.01∼−0.20, *p* < 0.05, *I*^2^ = 72.14%, respectively. However, the implicit memory performance to negative stimuli was similar between cMDD patients and controls (Effect size = 0.06, 95% CI = −0.30∼0.40, *p* = 0.74, *I*^2^ = 79.9%). For *r*MDD patients, the implicit memory to positive stimuli was still poorer than controls for Effect size: −0.80, 95% CI: −1.12 to −0.48; *p* < 0.001, *I*^2^ = 6.2% while reversed to negative stimuli for *r*MDD patients could recall more negative stimuli than controls (Effect size = 0.82, 95% CI: 0.51 to 1.13; *p* < 0.001, *I*^2^ = 0.0%). The performance to neutral stimuli was similar between patients and controls for Effect size = −0.24, 95% CI: −0.51 to 0.03; *p* = 0.08, *I*^2^ = 0.0%.

## 4. Discussion

### 4.1. Results summary

Present review mainly focused on examining the impairment of implicit memory in patients with current and remitted depression. Firstly, we conducted two initial meta-analyses to *c*MDD and *r*MDD patients separately to assess their general function of implicit memory. To further examine the implicit memory in detail, we categorized included studies into four groups based on the sub-function types of implicit memory (i.e., implicit learning group, positive, neutral, and negative groups of implicit memory bias) and conducted sub-group analysis to these groups.

The results of initial meta-analysis show that the general function of implicit memory in *c*MDD is impaired for Effect size = −0.30; 95% CI: −0.53 to −0.08; *p* < 0.001; *I*^2^ = 74.2%, but intact in *r*MDD for Effect size: −0.05, 95% CI: −0.32 to −0.22; *p* = 0.7; *I*^2^ = 0.0%. In subsequent sub-group analysis, *c*MDD patients are impaired to positive and neutral stimuli (Effect size = −0.66, 95% CI: −1.04 to −0.28; *p* < 0.05, *I*^2^ = 84.8% and Effect size = −0.60, 95% CI = −1.01∼−0.20, *p* < 0.05, *I*^2^ = 72.14%, respectively), but similar with controls to negative stimuli (Effect size = −0.17, 95% CI: −0.61 to 0.28; *p* = 0.46, *I*^2^ = 89.0%). For *r*MDD patients, their implicit memory to neutral stimuli was similar to controls (Effect size = −0.24, 95% CI: −0.51 to 0.03; *p* = 0.08, *I*^2^ = 0.0%), whereas the implicit memory was still abnormal when processing positive and negative stimuli: *r*MDD patients exhibited poorer and better performance (Effect size = 0.82, 95% CI: 0.51 to 1.13; *p* < 0.001, *I*^2^ = 0.0%) to positive and negative stimuli (Effect size = −0.80, 95% CI: −1.12 to −0.48; *p* < 0.001, *I*^2^ = 6.2%) accordingly compared to controls. In brief, the implicit memory was generally impaired in *c*MDD patients, except for the negative stimuli. For *r*MDD patients, the implicit memory was recovered to neutral stimuli, but still abnormal to positive and negative stimuli.

### 4.2. Implicit memory to neutral stimuli

The implicit memory to neutral stimuli was assessed through two aspects. The first aspect was recall performance of neutral stimuli. In these studies, participants need to recall stimuli that they have processed but not intentionally memorized before (e.g., (22, 23, 32, 35, 36)). The second aspect regarded the latent regularity as memory content (e.g., (37, 40–42)). Relevant studies usually adopted visual search paradigms and shared similar experiment designs and logic. Generally, all the search displays could be divided into repeated and random conditions. Unlike random condition, the repeated condition contains a valid but latent regularity that could predict target location across trials. The search efficiency in both two conditions can improve over time by practice effect, but will be more significant in repeated condition if participants could memorize the regularities. The present review found that the recall performance in cMDD patients was poorer than controls, but recovered in rMDD patients. Therefore, it indicated that the abnormality of implicit memory to neutral stimuli was caused by depressive episode, and would recover with remission.

It should be mentioned that, one study of Lamy et al. (41) applied a unique and different paradigm to measure the implicit memory to neutral stimuli. They adopted the contextual cueing effect task that developed firstly by Chun and Jiang (51). Different from implicit sequence learning and weather prediction that are commonly used in MDD studies, the distractors in contextual cueing task share similar saliency (e.g., shapes, color, or topology) with the target so that participants need to pay attention (or top-down attention) for searching the target and making response. That is, the expression of the contextual cueing effect would be affected by multiple factors, such as the participations of selective attention (52, 53), working memory (54, 55), and successful attentional guidance and response selection (56, 57). Thus, future studies need to verify the mechanism of absent contextual cueing effect in MDD patients, and whether it depends on the development of depression.

### 4.3. Implicit memory to positive and negative stimuli

For studies applied the positive and negative stimuli, they shared same procedure and logic with neutral stimuli. The subgroup analyses showed that *c*MDD patients performed poorer than controls, but similar to controls when recalling negative stimuli. This indicated that the negative-biased memory tendency in *c*MDD patients might compensate the general impairment of implicit memory. In other words, the implicit memory in patients was biased to negative information, and the bias made them perform well as controls while deficit to neutral stimuli. For *r*MDD patients, recall performance to positive and negative stimuli was still abnormal; *r*MDD patients recalled more negative and less positive stimuli. These results might reveal a stable (or trait) cognitive characteristic in MDD patients for they possessed negative-biased and positive-avoided memory tendencies. However, whether the abnormal implicit memory bias was associated with the development of depression is still unclear. Future studies could longitudinally examine the abnormal implicit memory tendency to figure out latent cognitive indicators of depression development.

### 4.4. Implicit memory paradigms

The implicit memory is one categorization of memory that could process without participation of the conscious. It is generally assessed through the repetition priming effect in various paradigms with reaction time (RT), accuracy (ACC), and number/percentage of recall as indicators. We summarized the paradigms that commonly used in MDD patients’ studies into two parts. The first part introduced the paradigms adopted emotional stimuli (i.e., positive, neutral, and negative); The second part focused on the paradigms that regarded latent regularities (e.g., spatial and semantic associations) as memory contents.

#### 4.4.1. Emotional paradigms

The emotional paradigms usually manipulated emotional types of stimuli, and were primarily used to examine the implicit memory bias to stimuli with different emotional types. In these studies, the stimuli were regarded as memory contents and categorized into different emotion types (e.g., positive, neutral and negative). As listed in Table 1, this function could be assessed through various tasks, such as Self-referent incidental recall (6, 7, 38, 47), Mental imagery (36, 39), and Word-stem completion task (21, 22, 33–35). Most of them shared one similar procedure: in study phase, the stimuli that consisted of words or pictures with different emotion types would present to participants one at time, and they were asked to pronounce, imagine, or make decisions (e.g., matching faces; whether the word describes themself; evaluating the emotion types or valences of stimuli) to each of the presented words. The requirements were designed to ensure that participants could process the semantics of each stimulus without intentional memory. In the test phase, participants were informed to recall stimuli they processed before, or recognize them freely (or in a set mixed with new stimuli). The implicit memory bias would be considered happen if the recall performance (recall, recognition, or fixation duration with eye-tracking) to specific stimuli is better in patients/controls compared to another group.

#### 4.4.2. Regularity paradigms

Each time we utilized the library to search for books of interests, the configuration of book categorizations and book placement within categorization we frequently browsed were stable. Such configurations helped us locate the target book more efficiently even though we have never intentionally memorized them. Psychologically, this improvement derived from repeated and stable configuration without conscious memory could be used to measure implicit memory.

Generally, most of these paradigms regarded regularities as memory contents and adopted various visual search tasks that participants will be asked to search specific targets in a series of search displays. Each display concluded one target and several distractors [e.g., single letter and geometric graphics, see (37, 41)]. All search displays could be divided into repeated and random conditions. In repeated condition, there was one or more regularities through trials, they were valid to predict the target location (or physical characteristics) so that the search performance would improve more efficiently than random condition, which has no such regularities. After completing the search task, participants usually needed to complete another recognition test to ensure that they were unconscious of the regularities in repeated condition. Tasks like implicit procedural learning (24, 37, 40, 42, 44, 45), and weather prediction (46, 48) tasks were commonly used. In an example of implicit procedural learning (40), participants need to judge which one of the four frames target would present in. All the search displays were categorized into repeated and random conditions. In repeated condition, the target location in each display was pseudo-random for the former target locations were associated with latter locations. Consequently, participants will perform better in repeated than random condition over time if they could implicitly memorize these location associations. Similarly, the weather prediction tasks (58) presented to participants with stimuli like words (*task 1*), geometric graphics (*task 2*), or artificial objects (*task 3*). Each display would be presented to participants with different combination patterns, and participants need to choose one of two possible outcomes while each combination pattern could predict the specific outcome with a different probability. Thus, the performance in each pattern should increase over time if participants could memorize the associations between combination patterns and outcomes. Lastly, Lamy et al. (41) adopted contextual cueing effect task that firstly adopted in Chun and Jiang (51) to assess patients’ implicit memory. In this task, participants are informed to search for a target letter ‘T’ among distractor letters ‘L’s in each display. The displays could be categorized into repeated and novel conditions for the locations of target and distractors within each search display are stable in the former, but random in the latter. Over time, participants would gain an advantage for searching for targets in repeated condition over novel condition.

## 5. Limitations and new insights

There are several major limitations: For the first, the heterogeneity between included studies was moderate to high. Thus, we conducted a random effects model throughout to provide a conservative estimate. The second is that the sample size in some studies is insufficient. For example, the number of MDD patients in the study of Aizenstein et al. (37) and Janacsek et al. (24) were 11 and 10. That make it hard to decrease the affection of extraneous variables, and insufficient in the statistical validation. In addition, the included studies were search based on three databases (i.e., PubMed, Web of Science, and EMBASE). It was possible that some other relevant studies might not be collected so that limited the final sample size. Lastly, we did not examine the possible associations between implicit memory and clinical characteristics (e.g., severity and recurrence) in MDD patients. Hence, future studies could include more studies with sufficient sample sizes and conduct more detailed sub-group analyses for a more elaborate and accurate investigation of the implicit memory impairment in MDD patients.

## 6. Conclusion

The primary purpose of the present review was to examine the elaborate function of implicit memory in MDD patients with different statuses (i.e., current and remission). The results of the initial meta-analysis revealed a general impairment of implicit memory in both *c*MDD and *r*MDD patients. Then, the following sub-group analyses showed that the implicit memory of positive and neutral stimuli was abnormal in *c*MDD patients. Notably, the implicit memory of negative stimuli in cMDD patients was intact as healthy controls. This may suggest a compensatory effect of negative-biased memory tendency to a general memory deficit in *c*MDD patients. Further, the implicit memory to neutral stimuli was recovered in *r*MDD patients but remained abnormal to positive and negative stimuli. This result suggested that the abnormality of implicit memory to positive and negative stimuli was stable, indicating that implicit memory’s positive-avoided and negative-biased tendencies might be a stable (or trait) dysfunction in MDD patients. Thus, we expect to provide a possible indicator (implicit memory to positive/negative stimuli) for the diagnosis and prediction of depression.

## Data availability statement

The raw data supporting the conclusions of this article will be made available by the authors, without undue reservation.

## Author contributions

XL: work design, data collecting, and manuscript writing. XW: technical and data collecting assistances. YL: data collecting assistance. FG, JX, and JF: make important revisions to this manuscript. XZ: guiding and co-ordinating the whole process. All authors contributed to the article and approved the submitted version.

## References

[1] Eaton WW Alexandre P Bienvenu OJ Clarke D Martins SS Zablotsky B. The burden of mental disorders. In: Eaton WW editor. *Public Mental Health.* New York, NY: Oxford University Press (2012). p. 3–30.

[2] Liu DY Thompson RJ. Selection and implementation of emotion regulation strategies in major depressive disorder: an integrative review. *Clin Psychol Rev.* (2017) 57:183–94. 10.1016/j.cpr.2017.07.004 28739277

[3] Chen C Jiang WH Wang W Ma XC Li Y Wu J Impaired visual, working, and verbal memory in first-episode, drug-naive patients with major depressive disorder in a Chinese population. *PLoS One.* (2018) 13:e0196023. 10.1371/journal.pone.0196023 29684091 PMC5912727

[4] Withall A Harris LM Cumming SR. The relationship between cognitive function and clinical and functional outcomes in major depressive disorder. *Psychol Med.* (2009) 39:393. 10.1017/S0033291708003620 18533056

[5] Callahan BL Simard M Mouiha A Rousseau F Laforce R Jr. Hudon C. Impact of depressive symptoms on memory for emotional words in mild cognitive impairment and late-life depression. *J Alzheimers Dis.* (2016) 52:451–62. 10.3233/JAD-150585 27031467

[6] Romero N Sanchez A Vázquez C Valiente C. Explicit self-esteem mediates the relationship between implicit self-esteem and memory biases in major depression. *Psychiatry Res.* (2016) 242:336–44. 10.1016/j.psychres.2016.06.003 27341330

[7] Vázquez C Diez-Alegria C Hernandez-Lloreda MJ Moreno MN. Implicit and explicit self-schema in active deluded, remitted deluded, and depressed patients. *J Behav Ther Exp Psychiatry.* (2008) 39:587–99. 10.1016/j.jbtep.2008.01.006 18339357

[8] Beck AT Rush AJ Shaw BF Emery G. *The Cognitive Therapy of Depression.* New York, NY: The Guilford Press (1979).

[9] Abramson LY Metalsky GI Alloy LB. Hopelessness depression: a theory-based subtype of depression. *Psychol Rev.* (1989) 96:358–72.

[10] Beck AT. Depression: clinical experimental, and theoretical aspects. *JAMA.* (1967) 203:1144–5. 10.1001/jama.1968.03140130056023

[11] Beck AT. The evolution of the cognitive model of depression and its neurobiological correlates. *Am J Psychiatry.* (2008) 165:969–77. 18628348 10.1176/appi.ajp.2008.08050721

[12] Bower GH. Mood and memory. *Am Psychol.* (1981) 36:129–48. 7224324 10.1037//0003-066x.36.2.129

[13] Ingram RE Miranda J Segal ZV. *Cognitive Vulnerability to Depression.* New York, NY: Guilford Press. (1998).

[14] Teasdale JD. Cognitive vulnerability to persistent depression. *Cogn Emot.* (1988) 2:247–74.

[15] Beevers CG. Cognitive vulnerability to depression: a dual process model. *Clin Psychol Rev.* (2005) 25:975–1002. 10.1016/j.cpr.2005.03.003 15905008

[16] Graf P Schacter DL. Implicit and explicit memory for new associations in normal in amnesic subjects. *J Exp Psychol Learn Mem Cogn.* (1985) 11:501–18. 10.1037//0278-7393.11.3.501 3160813

[17] MacLeod C Mathews AM. Cognitive–experimental approaches to the emotional disorders. In: Martin PR editor. *Handbook of Behavior Therapy and Psychological Science.* New York, NY: Pergamon Press (1991). p. 116–50.

[18] Mulligan NW. The effect of generation on long-term repetition priming in auditory and visual perceptual identification. *Acta Psychologica.* (2011) 137:18–23. 21388613 10.1016/j.actpsy.2011.02.001

[19] Neill WT. Episodic rerieval in negative priming and repetition priming. *J Exp Psychol Learn Mem Cogn.* (1997) 23:1291–3105.

[20] Schacter DL Chiu CYP Ochsner KN. Implicit Memory: a Selective Review. *Annu Rev Neurosci.* (1993) 16:159–82. 8460889 10.1146/annurev.ne.16.030193.001111

[21] Bazin N Perruchet P Feline A. Mood congruence effect in explicit and implicit memory tasks: a comparison between depressed patients, schizophrenic patients and controls. *Eur Psychiatry.* (1996) 11:390–5. 10.1016/s0924-9338(97)82575-8 19698488

[22] Danion JM Kauffmann-Muller F Grangé D Zimmermann MA Greth P. Affective valence of words, explicit and implicit memory in clinical depression. *J Affect Disord.* (1995) 34:227–34. 10.1016/0165-0327(95)00021-e 7560551

[23] Tarsia M Power MJ Sanavio E. Implicit and explicit memory biases in mixed anxiety-depression. *J Affect Disord.* (2003) 77:213–25. 10.1016/s0165-0327(02)00119-2 14612221

[24] Janacsek K Borbély-Ipkovich E Nemeth D Gonda X. How can the depressed mind extract and remember predictive relationships of the environment? Evidence from implicit probabilistic sequence learning. *Prog Neuropsychopharmacol Biol Psychiatry.* (2018) 81:17–24. 10.1016/j.pnpbp.2017.09.021 28958916

[25] O’Connor MG Jerskey BA Robertson EM Brenninkmeyer C Ozdemir E Leone AP. The effects of repetitive transcranial magnetic stimulation (rTMS) on procedural memory and dysphoric mood in patients with major depressive disorder. *Cogn Behav Neurol.* (2005) 18:223–7. 10.1097/01.wnn.0000187938.73918.33 16340396

[26] Hutton B Salanti G Caldwell DM Chaimani A Schmid CH Cameron C The PRISMA extension statement for reporting of systematic reviews incorporating network meta-analyses of health care interventions: checklist and explanations. *Ann Intern Med.* (2015) 162:777–84. 26030634 10.7326/M14-2385

[27] American Psychiatric Association [APA]. *Diagnostic and Statistical Manual of Mental Disorders: DSM-V.* 5th ed. Washington, DC: American Psychiatric Association (2013).

[28] World Health Organization [WHO]. *The International Classification of Diseases (ICD), 2nded.* Geneva: World Health Organization (1993).

[29] Wells G. *The Newcastle-Ottawa Scale (NOS) for Assessing the Quality of Non-Randomised Studies in Meta-Analyses. Paper presented at the Symposium on Systematic Reviews.* Milton Keynes, UK: Beyond the Basics (2014).

[30] Cohen J. *Statistical Power Analysis for the Behavioral Sciences.* 2nd ed. New York, NY: Lawrence Erlbaum Associates (1988).

[31] Higgins JP Thompson SG Deeks JJ Altman DG. Measuring inconsistency in meta-analyses. *BMJ.* (2003) 327:557–60. 12958120 10.1136/bmj.327.7414.557 PMC192859

[32] Elliott CL Greene RL. Clinical depression and implicit memory. *J Abnorm Psychol.* (1992) 101:572–4. 10.1037//0021-843x.101.3.572 1500615

[33] Bazin N Perruchet P De Bonis M Féline A. The dissociation of explicit and implicit memory in depressed patients. *Psychol Med.* (1994) 24:239–45. 10.1017/s0033291700027008 8208889

[34] Ilsley JE Moffoot APR O’Carroll RE. An analysis of memory dysfunction in major depression. *J Affect Disord.* (1995) 35:1–9. 10.1016/0165-0327(95)00032-I 8557882

[35] Watkins PC Martin CK Stern LD. Unconscious memory bias in depression: perceptual and conceptual processes. *J Abnorm Psychol.* (2000) 109:282–9. 10.1037//0021-843X.109.2.282 10895566

[36] Ellwart T Rinck M Becker ES. Selective memory and memory deficits in depressed inpatients. *Depress Anxiety.* (2003) 17:197–206. 10.1002/da.10102 12820175

[37] Aizenstein HJ Butters MA Figurski JL Stenger VA Reynolds ICF Carter CS. Prefrontal and striatal activation during sequence learning in geriatric depression. *Biol Psychiatry.* (2005) 58:290–6. 10.1016/j.biopsych.2005.04.023 16018981

[38] Lim SL Kim JH. Cognitive processing of emotional information in depression, panic, and somatoform disorder. *J Abnorm Psychol.* (2005) 114:50–61. 10.1037/0021-843x.114.1.50 15709812

[39] Rinck M Becker ES. A comparison of attentional biases and memory biases in women with social phobia and major depression. *J Abnorm Psychol.* (2005) 114:62–74. 10.1037/0021-843X.114.1.62 15709813

[40] Naismith SL Hickie IB Ward PB Scott E Little C. Impaired implicit sequence learning in depression: a probe for frontostriatal dysfunction? *Psychol Med.* (2006) 36:313–23. 10.1017/s0033291705006835 16359605

[41] Lamy D Goshen-Kosover A Aviani N Harari H Levkovitz H. Implicit memory for spatial context in depression and schizophrenia. *J Abnorm Psychol.* (2008) 117:954–61. 10.1037/a0013867 19025241

[42] Exner C Lange C Irle E. Impaired implicit learning and reduced pre-supplementary motor cortex size in early-onset major depression with melancholic features. *J Affect Disord.* (2009) 119:156–62. 10.1016/j.jad.2009.03.015 19345999

[43] Ridout N Dritschel B Matthews K McVicar M Reid IC O’Carroll RE. Memory for emotional faces in major depression following judgement of physical facial characteristics at encoding. *Cogn Emot.* (2009) 23:739–52. 10.1080/02699930802121137

[44] Pedersen A Kueppers K Behnken A Kroker K Schoening S Baune BT Implicit and explicit procedural learning in patients recently remitted from severe major depression. *Psychiatry Res.* (2009) 169:1–6. 10.1016/j.psychres.2008.06.001 19595464

[45] Naismith SL Lagopoulos J Ward PB Davey CG Little C Hickie IB. Fronto-striatal correlates of impaired implicit sequence learning in major depression: an fMRI study. *J Affect Disord.* (2010) 125:256–61. 10.1016/j.jad.2010.02.114 20219248

[46] Elderkin-Thompson V Moody T Knowlton B Hellemann G Kumar A. Explicit and implicit memory in late-life depression. *Am J Geriatric Psychiatry.* (2011) 19:364–73. 10.1097/JGP.0b013e3181e89a5b 20808121

[47] Romero N Sanchez A Vázquez C. Memory biases in remitted depression: the role of negative cognitions at explicit and automatic processing levels. *J Behav Ther Exp Psychiatry.* (2014) 45:128–35. 10.1016/j.jbtep.2013.09.008 24140810

[48] Mörkl S Blesl C Jahanshahi M Painold A Holl AK. Impaired probabilistic classification learning with feedback in patients with major depression. *Neurobiol Learn Mem.* (2016) 127:48–55. 10.1016/j.nlm.2015.12.001 26688109

[49] Nemeth V Csete G Drotos G Greminger N Janka Z Vecsei L The effect of emotion and reward contingencies on relational memory in major depression: an eye-movement study with follow-up. *Front Psychol.* (2016) 7:1849. 10.3389/fpsyg.2016.01849 27920752 PMC5118641

[50] Brian MC Jonathan PS Leah RK Elissa JH Lisa A O’Donnell C. Self-reported affective biases, but not all affective performance biases, are present in depression remission. *Br J Clin Psychol.* (2019) 58:274–88. 30854675 10.1111/bjc.12217 PMC6682436

[51] Chun MM Jiang Y. Contextual cueing: implicit learning and memory of visual context guides spatial attention. *Cogn Psychol.* (1998) 36:28–71. 10.1006/cogp.1998.0681 9679076

[52] Jiang YH Leung AW. Implicit learning of ignored visual context. *Psychon Bull Rev.* (2005) 12:100–6. 15948286 10.3758/bf03196353

[53] Jiménez L Vázquez GA. Implicit sequence learning and contextual cueing do not compete for central cognitive resources. *J Exp Psychol Hum Percept Perform.* (2011) 37:222. 10.1037/a0020378 20731503

[54] Manginelli AA Baumgartner F Pollmann S. Dorsal and ventral working memory-related brain areas support distinct processes in contextual cueing. *Neuroimage.* (2013) 67:363–74. 10.1016/j.neuroimage.2012.11.025 23201492

[55] Travis SL Mattingley JB Dux PE. On the role of working memory inspatial contextual cueing. *J Exp Psychol Learn Mem Cogn.* (2013) 39:208–19. 22642237 10.1037/a0028644

[56] Wang C Haponenko H Liu X Sun H Zhao G. How attentional guidance and response selection boost contextual learning: evidence from eye movement. *Adv Cogn Psychol.* (2019) 15:265–75. 10.5709/acp-0274-2 32477438 PMC7246933

[57] Zhao G Liu Q Jiao J Zhou P Li H Sun H. Dual-state modulation of the contextual cueing effect: evidence from eye movement recordings. *J Vis.* (2012) 12:11. 10.1167/12.6.11 22685338

[58] Knowlton BJ Squire LR Gluck MA. Probabilistic classification learning in amnesia. *Learn Mem.* (1994) 1:106–20. 10467589

