# Supplementary figures and images for: The abnormal implicit memory to positive and negative stimuli in patients with current and remitted major depressive disorder: A systematic review and meta-analysis
Source: Front Psychiatry. 2023 Jan 10;13:1043987. doi: 10.3389/fpsyt.2022.1043987 (PMC9871490; doi:10.3389/fpsyt.2022.1043987)

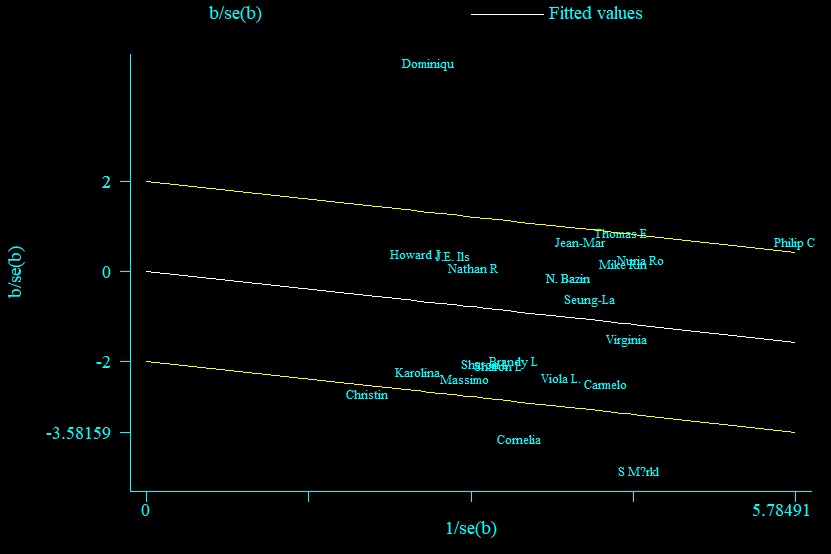

Supplement: Supplementary file 1 [file Image_1.JPEG]
